# Parity and the Risk of Diabetes Mellitus among Chinese Women: A Cross-Sectional Evidence from the Tongji-Dongfeng Cohort Study

**DOI:** 10.1371/journal.pone.0104810

**Published:** 2014-08-08

**Authors:** Yaohua Tian, Lijun Shen, Jing Wu, Weihong Chen, Jing Yuan, Handong Yang, Youjie Wang, Yuan Liang, Tangchun Wu

**Affiliations:** 1 MOE Key Lab of Environment and Health, School of Public Health, Tongji Medical College, Huazhong University of Science & Technology, Wuhan, China; 2 Department of Maternal and Child Health, School of Public Health, Tongji Medical College, Huazhong University of Science & Technology, Wuhan, China; 3 Dongfeng General Hospital, Dongfeng Motor Corporation and Hubei University of Medicine, Hubei, China; 4 Department of Social Medicine, School of Public Health, Tongji Medical College, Huazhong University of Science & Technology, Wuhan, China; Indiana University School of Medicine, United States of America

## Abstract

**Objectives:**

Little is known about the long-term health impact of pregnancy on women. The objective of this study was to examine the association between parity and the risk of diabetes among a population of Chinese women.

**Study design:**

A total of 14,196 women (aged ≥45 years) from the Dongfeng-Tongji Cohort study who had experienced at least one live birth completed baseline questionnaires, medical examinations, and provided baseline blood samples. Participants were categorized into four groups according to parity (one, two, three, or four or more live births). Logistic regression models were used to investigate the association between parity and the risk of diabetes after controlling potential confounders.

**Results:**

The prevalence of diabetes in the study population was 18.0% (2,552/14,196). Fasting plasma glucose levels increased with the increasing number of live births (*P*<0.001) and parity had a positive graded association with diabetes without adjustment for any covariates (*P* for trend <0.001). After adjusting for potential confounders, women who had had two, three, and four or more live births had 1.35 times (95% CI, 1.20–1.52), 1.59 times (95% CI, 1.39–1.82) and 1.44 times (95% CI, 1.21–1.71), respectively, higher risk of diabetes compared with women who had had one live birth.

**Conclusion:**

Multiparity was associated with increasing risk of diabetes in this population of Chinese women. These findings suggested that multiparity may be a risk factor for the development of diabetes among Chinese women. Future studies are needed to examine the physiological changes during pregnancy for risk of diabetes in later life.

## Introduction

The prevalence of diabetes is increasing because of aging, changes in lifestyle, and the increasing prevalence of obesity. In China, the age-standardized proportion of diabetes was as high as 9.7% [1], and the global prevalence of diabetes is estimated to reach 4.4% in 2030 [2]. Diabetes is closely associated with premature mortality and hospitalization for conditions such as cardiovascular and kidney diseases [3]. Pregnancy involves dramatic alterations in physiology, metabolism, and lifestyle, including a state of insulin resistance in peripheral tissues [4], weight gain or obesity, and postpartum weight retention [5], [6]. All these changes may have a long-term influence on the prospective health of women. Some studies have reported that pregnancy was associated with kidney diseases, hypertension, and autoimmune diseases [7], [8]. The relationship between parity and subsequent risk of diabetes has been a topic of research for many years, but the findings are inconsistent. Some studies have found that parity, particularly grand multiparity (five or more live births), showed a positive association with the incidence of diabetes [9]–[14]. However, other studies have found no relationship between parity and increased risk of diabetes [15], [16]. Some researchers have suggested that the relationship between parity and the incidence of diabetes observed in some studies may not be causal but could be confounded or mediated by other factors, such as adiposity or demographic factors [17].

The purpose of the current study was to examine whether the number of births is associated with the prevalence of diabetes in a population of Chinese women after controlling potential confounding factors, including lifestyle, and demographic and physiological factors. We hypothesized that parity was independently associated with the risk of diabetes in Chinese women.

## Materials and Methods

### Participants

The Dongfeng-Tongji Cohort (DFTJ cohort) study was launched in 2008 among retirees of Dongfeng Motor Corporation (DMC) in Shiyan City, Hubei Province. DMC was founded in 1969 and is one of the three largest auto manufactures in China. Wang F et al. previously described the design, fundamentals, and methods of the DFTJ cohort in detail [18]. Between 2008 and 2010, 87.0% (n = 27,009 out of 31,000) of retired employees who agreed to participate in the study were recruited and completed baseline questionnaires, medical examinations, and provided baseline blood samples.

Of the 27,009 eligible participants, 14,957 were women. We excluded women who had not had a live birth from our study (n = 205). We also excluded candidate participants whose information on parity or on diagnosis of diabetes was missing (n = 556). In total, 761 participants were excluded from our study (accounting for 5.1% of the population). The final sample size for the study was 14,196 women (mean age, 61.47 years).

The participants completed a reproductive questionnaire regarding the number of births or abortions, age at menopause, use of oral contraceptives, and hormone replacement therapy. Information on family history of diabetes was also obtained via questionnaire.

### Ethics Statement

Signed informed consent was obtained from all participants, and the study was approved by the Medical Ethics Committee of the School of Public Health, Tongji Medical College, and Dongfeng General Hospital.

### Assessment of diabetes mellitus

According to the American Diabetes Association criteria, individuals with diabetes mellitus included those self-reporting a physician diagnosis of diabetes; those under antidiabetic treatment; and those with high fasting plasma glucose (≥7.0 mmol/L), which was tested at baseline in this cohort study. As individuals on antidiabetic treatment might have normal fasting plasma glucose level despite having diabetes, they were excluded from the analysis of mean fasting plasma glucose (n = 1474). In the current study, we did not differentiate type 2 from type 1 diabetes mellitus. Nevertheless, more than 95% of all diagnosed diabetes is type 2 among older Chinese adults [19].

### Parity

Parity was defined as the self-reported total number of live births. Parity was classified into four categories: one live birth, two live births, three live births, and four or more live births.

### Assessment of covariates

Demographic information on sex, age, marital status (e.g., married, widowed, divorced, unmarried), and education (e.g., primary or below, junior high school, high school, college or above) was collected. Lifestyle information on physical activity, cigarette smoking status and alcohol drinking status was also obtained. Physical activity was defined as those who exercised more than 20 min per day and more than three times per week over the last 6 months. A positive family history of diabetes was defined as the self-report of at least one first-degree family member (father, mother, siblings, or offspring) being diagnosed by a physician as having diabetes. Height, weight, waist circumference, and systolic and diastolic blood pressures were measured with standard apparatus in the medical examinations. We calculated the body mass index by dividing weight in kilograms by height squared in meters.

### Statistical analysis

Categorical data were summarized as proportion (%) and numerical variables were summarized as means ± SD. Differences were analyzed with *t* tests or ANOVA for numerical variables and χ^2^ tests for categorical variables. We used diabetes mellitus as a dependent variable and a series of multivariate logistic regression models to calculate the odds ratios (OR) and 95% confidence intervals (95%CI) across parity categories. Because the outcomes of diabetes in older women had clearly exceeded 10%, the adjusted OR derived from logistic regression could not approximate the risk ratio. We used a formula recommended by Zhang *et al.* to correct the adjusted OR and 95% CI [20]. We used women with one birth as the reference category. Model 1 examined the relationship between parity and diabetes without adjustment for any covariates. Model 2 included education, marital status, cigarette smoking status, alcohol drinking status, physical activity, family history of diabetes, hypertension, and factors related to reproductive health including menopause status, oral contraceptive use, and hormone replacement therapy use. Model 3 included the covariates in model 2 plus abortion. Model 4 included the covariates in model 4 plus age. Finally, model 5, the fully adjusted model, included all the previously listed potential confounders in model 4 plus BMI, a known risk factor for diabetes. All statistical analyses were performed using SPSS software (version 17.0), and tests of statistical significance were set at *P*<0.05.

## Results

Table 1 presents the characteristics of the 14,196 participants according to parity categorized into four groups. Younger participants were more likely to have one child because of the one-child policy introduced in 1979. Age and BMI increased steadily across parity categories. Average fasting plasma glucose levels increased with increasing parity. Compared with women who had experienced one live birth, women with higher parity (two, three, and four or more live births) were more likely to report physician-diagnosed hypertension, to have lower education, and were less likely to have a family history of diabetes. In addition, a higher percentage of individuals with higher parity were smokers. Participants were less likely to use oral contraception with the increasing number of live births. The proportion of physical activity participation among different parity groups was not statistically significant.

**Table 1:** Baseline characteristics of study participants of 14196 women by parity.

	Parity					*P*
Characteristics	1(n = 4900)	2(n = 4766)	3(n = 2782)	≥4(n = 1748)	χ^2^/F	
Age (y) (mean ± SD)	55.28±4.98	61.38±5.70	66.33±6.17	71.28±6.16	4451.50	<0.001*
Marital status					780.94	<0.001‡
Unmarried (%)	7(0.1)	10(0.2)	5(0.2)	1(0.1)		
Married (%)	4448(91.0)	4167(87.8)	2316(83.3)	1266(72.5)		
Widowed (%)	236(4.8)	453(9.5)	429(15.4)	472(27.0)		
Divorced (%)	197(4.0)	117(2.5)	29(1.0)	7(0.4)		
Education					2805.85	<0.001‡
Elementary or below (%)	499(10.3)	1318(27.8)	1267(46.0)	1164(68.0)		
Junior high school (%)	1868(38.4)	1919(40.5)	967(35.1)	430(25.1)		
High school (%)	2001(41.1)	1145(24.2)	422(15.3)	96(5.6)		
College or above (%)	500(10.3)	354(7.5)	98(3.6)	22(1.3)		
Physical activity					0.70	0.873‡
Yes (%)	4323(88.2)	4194(88.0)	2466(88.6)	1542(88.2)		
Passive smoking					119.28	<0.001‡
Yes (%)	1216(24.8)	955(20.1)	481(17.3)	245(14.0)		
Current smoker					178.80	<0.001‡
Yes (%)	48(1.0)	82(1.7)	75(2.7)	111(6.4)		
Current alcohol drinker					10.28	0.016 ‡
Yes (%)	342(7.0)	282(5.9)	149(5.4)	98(5.6)		
Family history of DM					201.79	<0.001‡
Yes (%)	536(11.1)	267(5.7)	116(4.2)	63(3.6)		
Menopause status					1109.54	<0.001‡
Yes (%)	3910(79.9)	4549(95.6)	2715(97.6)	1716(98.2)		
Ever used Contraceptives					77.32	<0.001‡
Yes (%)	1225(25.5)	1206(25.4)	611(22.0)	275(15.8)		
Ever used hormone replacement					38.99	<0.001‡
Yes (%)	191(3.9)	160(3.4)	58(2.1)	24(1.4)		
Hypertension					614.51	<0.001‡
Yes (%)	1958(40.0)	2510(52.7)	1767(63.5)	1192(68.2)		
Abortion	1.33±1.20	1.08±1.12	0.92±1.07	0.76±1.03	141.62	<0.001*
BMI(kg/m^2^)	23.85±3.31	24.56±3.43	25.20±3.62	25.20±3.91	115.08	<0.001*

Note Abbreviations: y, years; DM, diabetes mellitus, BMI, body mass index.

Data are means ± SD or n (%) unless otherwise indicated.

*ANVOA test numerical data.

‡ χ^2^ test for categorical data.

Table 2 presents the average fasting plasma glucose levels according to parity categories. Average fasting plasma glucose levels increased steadily across parity categories, ranging from 5.76±1.50 mmol/L among women who had experienced one live birth to 6.01±1.68 mmol/L among women who had experienced two live births, 6.23±1.75 mmol/L among women who had experienced three live births, and 6.27±1.86 mmol/L among women who had experienced four or more live births. Post hoc analysis showed that women with higher parity (parity = two births, *P*<0.001; parity = three births, *P*<0.001; and parity≥four births, *P*<0.001) had higher fasting plasma glucose levels than women who had experienced one live birth. The Spearman correlation analysis showed that level of fasting plasma glucose was positive related to the number of parity (r = 0.15, *P*<0.001). Of the total 14,196 eligible study participants, 2,552 (18.0%) had diabetes, including those reporting a diagnosis by a physician, those with fasting plasma glucose higher than 7.0 mmol/L as measured in this study, and those taking antidiabetic medication. The prevalence of diabetes ranged from 11.1% in women who had experienced one live birth to 18.0% in women who had had two live births, 24.8% in women who had had three live births, and 26.2% in women who had had four or more live births. Unadjusted and multivariate adjusted OR and 95% CI are presented in Table 3. Because the use of OR was less justified in our study where outcomes of diabetes exceeded 10% [20], [21], the risk ratio (RR) and 95% CI were estimated using a formula [20] to correct the adjusted OR obtained from logistic regression.

**Table 2:** Parity and the level of fasting plasma glucose.

Parity	N	Fasting plasma glucose(mmol/L)	F	P
1	4649	5.76±1.50	58.28	<0.001
2	4279	6.01±1.68*		
3	2349	6.23±1.75*		
≥4	1445	6.27±1.86*		

Note *P<0.05 for the comparison with women with one live birth, by using ANVOA and Dunnet’s test for *post hoc* analysis.

**Table 3:** Unadjusted and adjusted odd ratios and risk ratios (RRs) (95%CI) for diabetes by parity.

Adjustments	Parity	*P*	OR	RR
Model 1: unadjusted	1		1	1
	2	<0.001	1.76(1.57–1.97)	1.62(1.48–1.78)
	3	<0.001	2.64(2.33–2.98)	2.23(2.03–2.44)
	≥4	<0.001	2.84(2.47–3.26)	2.36(2.12–2.61)
Test for trend‡				*P*<0.001
Model 2: Basic model*	1		1	1
	2	<0.001	1.65(1.45–1.88)	1.54(1.38–1.71)
	3	<0.001	2.29(1.98–2.64)	2(1.79–2.23)
	≥4	<0.001	2.27(1.92–2.68)	1.99(1.74–2.26)
Test for trend‡				*P*<0.001
Model 3: Model 2 factors + Abortion	1			1
	2	<0.001	1.63(1.44–1.86)	1.52(1.37–1.70)
	3	<0.001	2.25(1.95–2.59)	1.98(1.76–2.20)
	≥4	<0.001	2.22(1.88–2.63)	1.96(1.71–2.23)
Test for trend‡				*P*<0.001
Model 4: model 3 factors + Age	1		1	1
	2	<0.001	1.45(1.27–1.66)	1.38(1.23–1.55)
	3	<0.001	1.8(1.53–2.11)	1.65(1.44–1.88)
	≥4	<0.001	1.61(1.31–1.96)	1.51(1.27–1.77)
Test for trend‡				*P*<0.001
Model 5: model 4 factors + BMI	1		1	1
	2	<0.001	1.41(1.23–1.62)	1.35(1.20–1.52)
	3	<0.001	1.72(1.46–2.03)	1.59(1.39–1.82)
	≥4	<0.001	1.52(1.24–1.87)	1.44(1.21–1.71)
Test for trend				*P*<0.001

Note *Basic model: adjusted for education, marital status, passive smoking status, smoking status, alcohol drinking status, family history of DM, physical activity, hypertension, menopause status, ever use of contraceptives, and ever use of hormone replacement therapy.

Risk ratios calculated by using the formula (RR = OR/((1−P_0_)+(P_0_×OR)), P_0_: prevalence of diabetes in women with one live birth) to correct the adjusted OR above.

Abbreviations: BMI, body mass index.

‡ Cochran-Armitage test for trend.

We estimated four logistic regression models for the evaluation of the relationship between parity and risk of diabetes to control the major confounders of diabetes (Table 3). Model 1 showed that women who had had two live births (RR, 1.62; 95% CI, 1.48–1.78), or three live births (RR, 2.23; 95% CI, 2.03–2.44), or four or more live births (RR, 2.36; 95% CI, 2.12–2.61) had a significantly higher risk of diabetes in the unadjusted analysis compared with women who had had one live birth. The results of Model 2 showed that higher parity (two live births: RR, 1.54; 95% CI, 1.38–1.71; three live births: RR, 2.00; 95% CI, 1.79–2.23; and four or more live births: RR, 1.99; 95% CI, 1.74–2.26) was associated with an increased risk of diabetes, after adjustment for education, marriage, passive smoking status, smoking status, alcohol drinking status, family history of diabetes, physical activity, hypertension, menopause status, lifetime use of contraceptives, and lifetime use of hormone replacement therapy. These covariates were reported to be associated with the risk of diabetes [22]–[26]. As abortion has a similar biological process as live birth [25], we added it into model 3, and we still observed a significantly higher risk in women with higher parity after adjustment for abortion. In Model 4, after an additional adjustment for age which is a major risk factor for diabetes [27] and is associated closely with number of parity in our study, RR attenuated but remained statistically significant, ranging from 1.38 (95% CI, 1.23–1.55) in women who had experienced two live births to 1.65 (95% CI, 1.44–1.88) in women who had experienced three live births, and 1.51 (95% CI, 1.27–1.77) in women who had experienced four or more live births. In the fully adjusted model (Model 5), which included BMI, a main known risk factor for diabetes [22], [23], the RR were 1.35 (95% CI, 1.20–1.52), 1.59 (95% CI, 1.39–1.82), and 1.44 (95% CI, 1.21–1.71) for women who had experienced two, three, and four or more live births, respectively, compared with women who had experienced one live birth. The results of Cochran-Armitage test for trend showed that RRs were increased with the number of parity (All *P*s<0.001, Table 3).

## Discussion

To the best of our knowledge, this is the first study investigating the association between parity and risk of diabetes in a population of women in mainland China. We found that fasting plasma glucose levels and the prevalence of diabetes were associated with the number of live births. After adjusting for potential confounders, the association between parity and risk of diabetes was attenuated but remained statistically significant, suggesting that higher parity was a risk factor for diabetes and higher fasting blood glucose in this population of Chinese women.

In our study, we defined women who had had one live birth as the reference group. Nulliparous women typically have significant differences in lifestyle and physiology, compared with parous women [28], [29], and nulliparity is mainly because of polycystic ovary disease among Chinese women [30], a known risk factor for diabetes. Furthermore, nulliparity may be associated with diabetes because of underlying insulin resistance and β-cell dysfunction [31]. Therefore, we excluded all nulliparous women from data analysis.

The findings of the current study are consistent with most previous studies on this topic. For example, Kritz-Silverstein et al. [14] examined the relationship between parity and prevalence of diabetes in a population-based study of 1,186 women aged 41–92 years, and found that the risk of diabetes increased slightly with the number of live births independent of age, BMI, and family history of diabetes. A recent study conducted by Mueller et al. [11] examined the association between parity and diabetes among 25,021 Singapore Chinese women aged 45–74 years, and found a positive association between parity and risk of diabetes before and after adjustment for demographics, lifestyle behaviors, reproductive health factors, and BMI. However, they did not directly investigate the association between parity and fasting plasma glucose levels. Most other studies demonstrated that only grand multiparous women (parity: five or more) had a significant risk of developing diabetes. Nicholson et al. [12] reported that women who had experienced five or more live births had a 27% increased risk for diabetes after adjustment for covariates in a prospective cohort study of 7,024 Caucasian and African-American women. In the present study, we observed that the risk of diabetes increased in each increasing parity group.

The mechanism underlying the link between parity and diabetes is unclear. Pregnancy involves dramatic alterations in physiology, metabolism and lifestyle. A state of insulin resistance in peripheral tissues is induced during pregnancy because of changes in some diabetogenic hormones and cortisol, including high levels of placental growth hormone, placental lactogen, circulating insulin-like growth factor I, gestational hormones, and tumor necrosis factor-α [4], [32], [33]. The β-cell mass expands to adapt to the progressive insulin resistance and insulin secretion increases to maintain normal blood sugar levels during pregnancy and the postpartum period [34], [35]. This metabolic stress may exhaust β-cells, leading to insulin secretion dysfunction and the development of diabetes mellitus later in life.

Pregnancy is an important period for women, and may result in postpartum weight retention and obesity, which are of crucial importance to a woman’s health in the future [5], [6]. It is a custom to attach great importance to dietary nutrition and the safety of pregnant women in China. For example, Chinese women during pregnancy are not allowed to participate in almost any form of physical activity so as to avoid the possibility of accidents. In addition, according to traditional Chinese practices, women should be confined to bed for a month after childbirth [36]. Lack of exercise and a high-calorie diet are closely related to an increased risk of weight gain or obesity, an important risk factor for diabetes in later life [37], [38]. It was reported that the average pregnancy weight gain was 17.1±4.9 kg among 16,460 Chinese women [39], which is much higher than that recommended [40]. The association between parity and the incidence of diabetes observed in some studies may be explained by obesity [41], [42]. In our study, however, the association was attenuated but remained statistically significant after adjustment for BMI. However, a limitation is that we measured BMI after the development of diabetes. The conclusion of the study would be more persuasive if BMI measured just after delivery had been used as a covariate [15].

Parity is a well-established protective factor for breast cancer [43]. However, accumulating evidence suggests that parity is associated with a higher risk of all-cause mortality later in life, and especially with cardiovascular and cerebrovascular mortality [44]. As diabetes is a major underlying cause of cardiovascular disease, the relationship between parity and diabetes observed in our study might explain the association between parity and cardiovascular and cerebrovascular mortality to some extent.

Our study has several strengths. With standardized questionnaires, laboratory measures, and standardized measures of several potential confounders, information regarding demographics, lifestyle behavior, history of disease, and reproductive health factors were available and considered valid in our study. In particular, levels of fasting plasma glucose were measured with unified instruments at several hospitals belonging to DMC. Therefore, ascertainment bias was unlikely in our study.

Our study also has some limitations. First, our data were cross-sectional. Although we demonstrated that higher parity was associated with risk of diabetes in this study, causal and temporal associations could not be inferred. However, the mean age at diagnosis of diabetes among women with self-reported diabetes was 55.4 years in this study, and the mean age for childbirth in China was 28.2 years [45]. Therefore, we could infer that the majority of women in our study had completed their childbearing before developing diabetes. In our view, parity is likely a causal factor for the development of diabetes. Second, we did not include pregnancy-related factors, such as history of gestational diabetes, maternal weight gain during pregnancy, or postpartum weight retention, which are risk factors for developing diabetes in later life [15].

In conclusion, parity is associated with the risk of diabetes independent of potential confounders. The association may be mediated in part by higher BMI but is not fully explained by adiposity. Additional longitudinal studies, in which a history of gestational diabetes, weight gain measures, and changes in physiology and lifestyle during pregnancy and the postpartum period can be prospectively measured, should be conducted to confirm the findings.
